# Supplementary material for: NK Cells Lose Their Cytotoxicity Function against Cancer Stem Cell-Rich Radiotherapy-Resistant Breast Cancer Cell Populations
Source: Int J Mol Sci. 2021 Sep 6;22(17):9639. doi: 10.3390/ijms22179639 (PMC8431804; doi:10.3390/ijms22179639)

<Supplementary Materials: Figure S1>

Raw images of western blotting (1/3)

Figure 2A

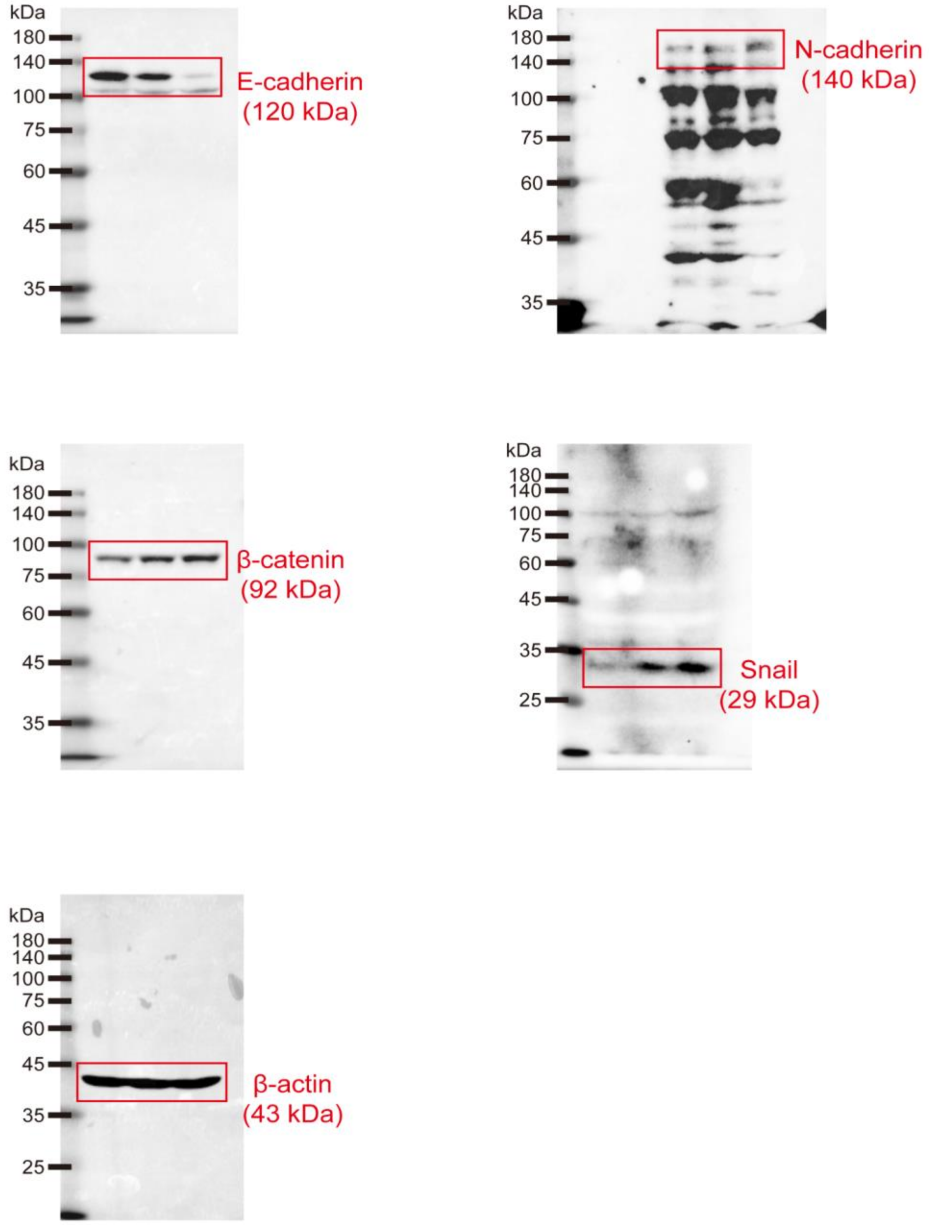

Raw images of western blotting (2/3)

Figure 2B

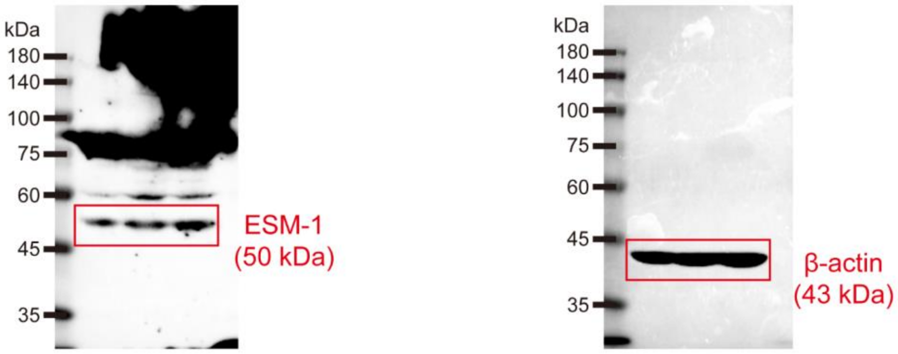

Figure 2C

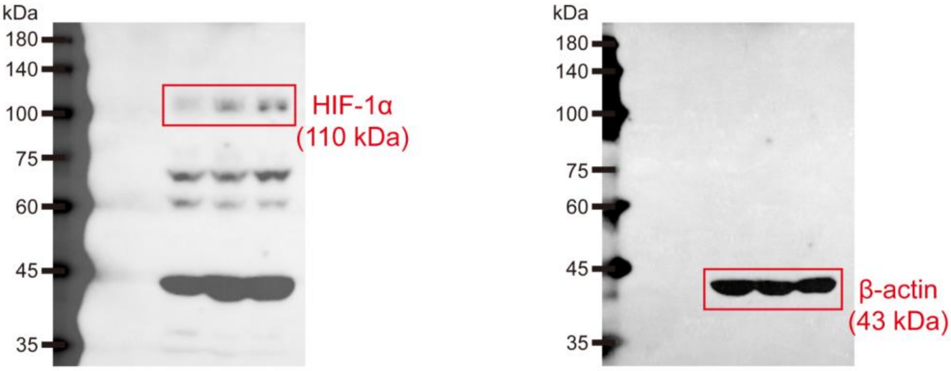

Figure 2D

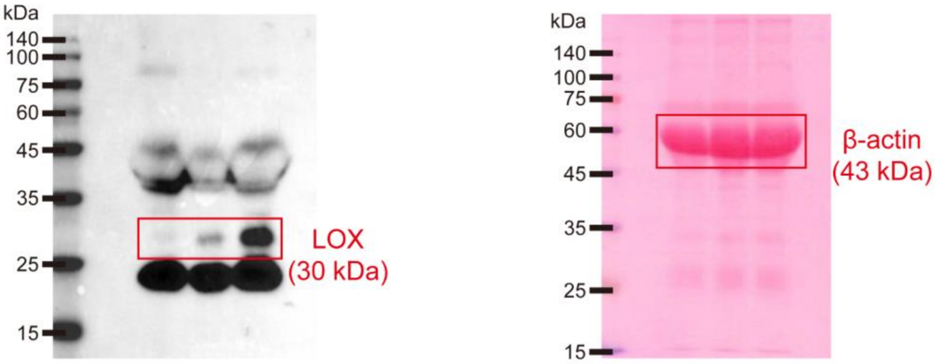

Raw images of western blotting (3/3)

Figure 4B

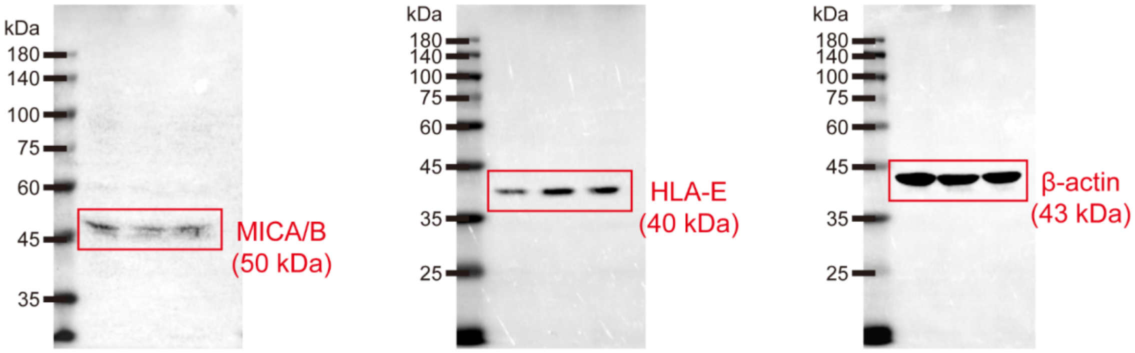

Figure 4C

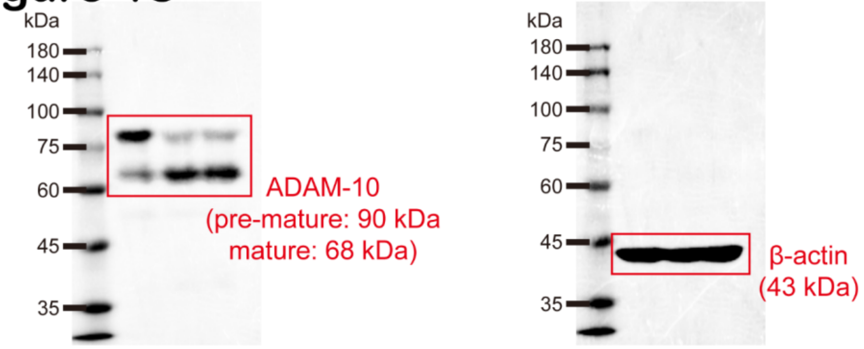

Supplement: Supplementary file 1 [file ijms-22-09639-s001.zip › Supplemental Materials-Figure S1.pdf]
