# Supplementary material for: NK Cells Lose Their Cytotoxicity Function against Cancer Stem Cell-Rich Radiotherapy-Resistant Breast Cancer Cell Populations
Source: Int J Mol Sci. 2021 Sep 6;22(17):9639. doi: 10.3390/ijms22179639 (PMC8431804; doi:10.3390/ijms22179639)
Supplement: Supplementary file 1 [file ijms-22-09639-s001.zip › Supplemental Materials-Tables.pdf]

<Table S1: Intensity ratios of Western blots for Figure 2A>

Fig 2A (E-cadherin)	n=1	E-cadherin	β-actin	E-cad/β-actin	Fold of MDA-MB-231
	**MDA-MB-231**	45767.321	55287.401	0.8278	1
	**RT-R-MDA-MB-231**	22469.480	46895.338	0.4791	0.5788
	**CD24-44+**	27330.522	48287.380	0.5660	0.6837
	**n=2**	**E-cadherin**	**β-actin**	**E-cad/β-actin**	**Fold of MDA-MB-231**
	**MDA-MB-231**	42015.714	61088.865	0.6878	1
	**RT-R-MDA-MB-231**	36957.785	61730.258	0.5987	0.8705
	**CD24-44+**	32897.037	59652.409	0.5515	0.8018
	**n=3**	**E-cadherin**	**β-actin**	**E-cad/β-actin**	**Fold of MDA-MB-231**
	**MDA-MB-231**	53970.551	60378.803	0.8939	1
	**RT-R-MDA-MB-231**	36683.794	60536.338	0.6060	0.6779
	**CD24-44+**	14578.912	64575.823	0.2258	0.2526
	**n=4**	**E-cadherin**	**β-actin**	**E-cad/β-actin**	**Fold of MDA-MB-231**
	**MDA-MB-231**	52468.271	56346.844	0.9312	1
	**RT-R-MDA-MB-231**	19855.045	57322.380	0.3464	0.3720
	**CD24-44+**	28996.321	57471.823	0.5045	0.5418

n=5	E-cadherin	β-actin	E-cad/β-actin	Fold of MDA-MB-231
**MDA-MB-231**	43543.200	56302.359	0.7734	1
**RT-R-MDA-MB-231**	35741.028	54175.397	0.6597	0.8530
**CD24-44+**	14021.296	59922.480	0.2340	0.3026

Fig 2A (N-cadherin)

n=1	N-cadherin	β-actin	N-cad/β-actin	Fold of MDA-MB-231
**MDA-MB-231**	25736.501	65639.945	0.3921	1
**RT-R-MDA-MB-231**	37438.907	62934.116	0.5949	1.5172
**CD24-44+**	41942.886	40010.095	1.0483	2.6737

n=2	N-cadherin	β-actin	N-cad/β-actin	Fold of MDA-MB-231
**MDA-MB-231**	14695.309	57031.844	0.2577	1
**RT-R-MDA-MB-231**	24326.137	57764.137	0.4211	1.6344
**CD24-44+**	51826.108	53090.409	0.9762	3.7885

n=3	N-cadherin	β-actin	N-cad/β-actin	Fold of MDA-MB-231
**MDA-MB-231**	16019.681	27099.146	0.5912	1
**RT-R-MDA-MB-231**	33866.522	24484.527	1.3832	2.3398
**CD24-44+**	51878.693	30985.953	1.6743	2.8322

n=4	N-cadherin	β-actin	N-cad/β-actin	Fold of MDA-MB-231
**MDA-MB-231**	6922.459	65362.430	0.1059	1
**RT-R-MDA-MB-231**	10355.066	49150.820	0.2107	1.9893
**CD24-44+**	21460.087	56317.953	0.3811	3.5979

n=5	N-cadherin	β-actin	N-cad/β-actin	Fold of MDA-MB-231
**MDA-MB-231**	15172.108	66518.066	0.2281	1
**RT-R-MDA-MB-231**	26842.886	55259.518	0.4858	2.1297
**CD24-44+**	50044.007	56318.167	0.8886	3.8958

Fig 2A (β-catenin)

n=1	β-catenin	β-actin	β-catenin/β-actin	Fold of MDA-MB-231
**MDA-MB-231**	24582.723	55327.693	0.4443	1
**RT-R-MDA-MB-231**	41331.057	46662.874	0.8857	1.9935
**CD24-44+**	51727.421	50972.258	1.0148	2.2840

n=2	β-catenin	β-actin	β-catenin/β-actin	Fold of MDA-MB-231
**MDA-MB-231**	25808.108	64290.087	0.4014	1
**RT-R-MDA-MB-231**	43122.158	56463.602	0.7637	1.9025
**CD24-44+**	36495.158	45629.945	0.7998	1.9924

n=3	β-catenin	β-actin	β-catenin/β-actin	Fold of MDA-MB-231
**MDA-MB-231**	22769.744	58775.874	0.3874	1
**RT-R-MDA-MB-231**	49375.187	59512.773	0.8297	2.1416
**CD24-44+**	47777.208	62288.602	0.7670	1.9799

n=4	β-catenin	β-actin	β-catenin/β-actin	Fold of MDA-MB-231
**MDA-MB-231**	31223.865	56233.602	0.5553	1
**RT-R-MDA-MB-231**	41118.735	54054.794	0.7607	1.3700
**CD24-44+**	48234.401	53035.116	0.9095	1.6380

n=5	β-catenin	β-actin	β-catenin/β-actin	Fold of MDA-MB-231
**MDA-MB-231**	24630.815	45544.430	0.5408	1
**RT-R-MDA-MB-231**	33480.815	31976.388	1.0470	1.9361
**CD24-44+**	54002.401	47527.744	1.1362	2.1010

Fig 2A (Snail)	n=1	Snail	β-actin	Snail/β-actin	Fold of MDA-MB-231
	**MDA-MB-231**	15274.744	52206.936	0.2926	1

**RT-R-MDA-MB-231**	39713.007	46285.459	0.8580	2.9325
**CD24-44+**	54773.915	50659.844	1.0812	3.6954

**n=2**	**Snail**	**β-actin**	**Snail/β-actin**	**Fold of MDA-MB-231**
**MDA-MB-231**	16262.853	64087.794	0.2538	1
**RT-R-MDA-MB-231**	36306.167	57192.137	0.6348	2.5016
**CD24-44+**	53285.966	46197.238	1.1534	4.5454

**n=3**	**Snail**	**β-actin**	**Snail/β-actin**	**Fold of MDA-MB-231**
**MDA-MB-231**	6144.033	60042.752	0.1023	1
**RT-R-MDA-MB-231**	13648.024	59736.581	0.2285	2.2327
**CD24-44+**	20122.966	64056.945	0.3141	3.0700

**n=4**	**Snail**	**β-actin**	**Snail/β-actin**	**Fold of MDA-MB-231**
**MDA-MB-231**	10433.731	56754.016	0.1838	1
**RT-R-MDA-MB-231**	31038.489	57634.844	0.5385	2.9294
**CD24-44+**	25653.095	53190.652	0.4823	2.6234

**n=5**	**Snail**	**β-actin**	**Snail/β-actin**	**Fold of MDA-MB-231**
**MDA-MB-231**	2621.033	53944.894	0.0486	1
**RT-R-MDA-MB-231**	2641.882	41766.004	0.0633	1.3019

	**CD24-44+**	14679.087	57376.551	0.2558	5.2655

<Table S2: Summary of relative protein levels for Figure 2A>

**E-cadherin**	**MDA-MB-231**	**RT-R-MDA-MB-231**	**CD24-44+**
n=1	1	0.5788	0.6837
n=2	1	0.8705	0.8018
n=3	1	0.6779	0.2526
n=4	1	0.3720	0.5418
n=5	1	0.8530	0.3026
**Mean**	**1**	**0.6704**	**0.5165**
**Std. Deviation**	**0**	**0.2067**	**0.2374**
**Std. Error of Mean**	**0**	**0.0924**	**0.1062**

**N-cadherin**	**MDA-MB-231**	**RT-R-MDA-MB-231**	**CD24-44+**
n=1	1	1.5172	2.6737
n=2	1	1.6344	3.7885
n=3	1	2.3398	2.8322
n=4	1	1.9893	3.5979
n=5	1	2.1297	3.8958
**Mean**	**1**	**1.9220**	**3.3580**
**Std. Deviation**	**0**	**0.3423**	**0.5650**

**Std. Error of Mean**	**0**	**0.1531**	**0.2527**

**β-catenin**	**MDA-MB-231**	**RT-R-MDA-MB-231**	**CD24-44+**
n=1	1	1.9935	2.2840
n=2	1	1.9025	1.9924
n=3	1	2.1416	1.9799
n=4	1	1.3700	1.6380
n=5	1	1.9361	2.1010
**Mean**	**1**	**1.8690**	**1.9990**
**Std. Deviation**	**0**	**0.2935**	**0.2358**
**Std. Error of Mean**	**0**	**0.1312**	**0.1055**

**Snail**	**MDA-MB-231**	**RT-R-MDA-MB-231**	**CD24-44+**
n=1	1	2.9325	3.6954
n=2	1	2.5016	4.5454
n=3	1	2.2327	3.0700
n=4	1	2.6234	2.9294
n=5	1	1.3019	5.2655
**Mean**	**1**	**2.3180**	**3.9010**
**Std. Deviation**	**0**	**0.6214**	**0.9943**
**Std. Error of Mean**	**0**	**0.2779**	**0.4446**

<Table S3: Intensity ratios of Western blots for Figure 2B>

Fig 2B (ESM-1)	n=1	ESM-1	β-actin	ESM-1/β-actin	Fold of MDA-MB-231
	**MDA-MB-231**	16543.643	61480.865	0.2691	1
	**RT-R-MDA-MB-231**	28633.886	31879.569	0.8982	3.3379
	**CD24-44+**	37949.371	33287.853	1.1400	4.2367

n=2	ESM-1	β-actin	ESM-1/β-actin	Fold of MDA-MB-231
**MDA-MB-231**	12344.966	45040.782	0.2741	1
**RT-R-MDA-MB-231**	41074.785	46524.740	0.8829	3.2211
**CD24-44+**	57760.279	51894.581	1.1130	4.0609

n=3	ESM-1	β-actin	ESM-1/β-actin	Fold of MDA-MB-231
**MDA-MB-231**	16845.593	55656.309	0.3027	1
**RT-R-MDA-MB-231**	36944.836	56165.974	0.6578	2.1732
**CD24-44+**	46886.865	59165.066	0.7925	2.6183

n=4	ESM-1	β-actin	ESM-1/β-actin	Fold of MDA-MB-231
**MDA-MB-231**	23422.258	58109.037	0.4031	1
**RT-R-MDA-MB-231**	34861.472	52210.731	0.6677	1.6565
**CD24-44+**	43870.907	54081.602	0.8112	2.0125

	n=5	ESM-1	β-actin	ESM-1/β-actin	Fold of MDA-MB-231
	**MDA-MB-231**	16392.794	48317.543	0.3393	1
	**RT-R-MDA-MB-231**	32523.836	41266.380	0.7881	2.3230
	**CD24-44+**	54836.865	50078.087	1.0950	3.2276

<Table S4: Summary of relative protein levels for Figure 2B>

ESM-1	MDA-MB-231	RT-R-MDA-MB-231	CD24-44+
n=1	1	3.3379	4.2367
n=2	1	3.2211	4.0609
n=3	1	2.1732	2.6183
n=4	1	1.6565	2.0125
n=5	1	2.3230	3.2276
**Mean**	**1**	**2.5420**	**3.2310**
**Std. Deviation**	**0**	**0.7181**	**0.9434**
**Std. Error of Mean**	**0**	**0.3211**	**0.4219**

<Table S5: Intensity ratios of Western blots for Figure 2C>

Fig 2C (HIF-1α)	n=1	HIF-1α	β-actin	HIF-1α/β-actin	Fold of MDA-MB-231
	**MDA-MB-231**	2660.477	46163.007	0.0576	1
	**RT-R-MDA-MB-231**	6223.104	31593.250	0.1970	3.4178
	**CD24-44+**	7677.326	44587.915	0.1722	2.9876
	**n=2**	**HIF-1α**	**β-actin**	**HIF-1α/β-actin**	**Fold of MDA-MB-231**
	**MDA-MB-231**	6800.891	35559.359	0.1913	1
	**RT-R-MDA-MB-231**	16161.459	43679.359	0.3700	1.9346
	**CD24-44+**	33679.087	50704.238	0.6642	3.4730
	**n=3**	**HIF-1α**	**β-actin**	**HIF-1α/β-actin**	**Fold of MDA-MB-231**
	**MDA-MB-231**	6200.983	50331.388	0.1232	1
	**RT-R-MDA-MB-231**	14566.974	64547.368	0.2257	1.8318
	**CD24-44+**	23620.009	61703.803	0.3828	3.1070
	**n=4**	**HIF-1α**	**β-actin**	**HIF-1α/β-actin**	**Fold of MDA-MB-231**
	**MDA-MB-231**	6677.376	50286.723	0.1328	1
	**RT-R-MDA-MB-231**	18680.401	48428.581	0.3857	2.9049
	**CD24-44+**	27919.815	54597.652	0.5114	3.8511

	n=5	HIF-1α	β-actin	HIF-1α/β-actin	Fold of MDA-MB-231
	**MDA-MB-231**	15892.844	60732.401	0.2617	1
	**RT-R-MDA-MB-231**	39933.442	52109.459	0.7663	2.9285
	**CD24-44+**	48503.350	55090.823	0.8804	3.3644

<Table S6: Summary of relative protein levels for Figure 2C>

HIF-1α	MDA-MB-231	RT-R-MDA-MB-231	CD24-44+
n=1	1	3.4178	2.9876
n=2	1	1.9346	3.4730
n=3	1	1.8318	3.1070
n=4	1	2.9049	3.8511
n=5	1	2.9285	3.3644
**Mean**	**1**	**2.6040**	**3.3570**
**Std. Deviation**	**0**	**0.6897**	**0.3378**
**Std. Error of Mean**	**0**	**0.3084**	**0.1511**

<Table S7: Intensity ratios of Western blots for Figure 2D>

Fig 2D (LOX)

n=1	LOX	β-actin	LOX/β-actin	Fold of MDA-MB-231
**MDA-MB-231**	12490.698	50140.530	0.2491	1
**RT-R-MDA-MB-231**	57525.602	63226.246	0.9098	3.6523
**CD24-44+**	56879.581	54361.966	1.0463	4.2001

n=2	LOX	β-actin	LOX/β-actin	Fold of MDA-MB-231
**MDA-MB-231**	5601.305	50681.309	0.1105	1
**RT-R-MDA-MB-231**	10862.924	59539.217	0.1824	1.6508
**CD24-44+**	47638.622	62669.894	0.7602	6.8779

n=3	LOX	β-actin	LOX/β-actin	Fold of MDA-MB-231
**MDA-MB-231**	12750.971	45891.329	0.2779	1
**RT-R-MDA-MB-231**	59094.693	66753.137	0.8853	3.1861
**CD24-44+**	47350.803	49860.995	0.9497	3.4179

n=4	LOX	β-actin	LOX/β-actin	Fold of MDA-MB-231
**MDA-MB-231**	14036.167	45521.894	0.3083	1
**RT-R-MDA-MB-231**	16632.844	36585.974	0.4546	1.4744
**CD24-44+**	45204.099	49359.128	0.9158	2.9702

	n=5	LOX	β-actin	LOX/β-actin	Fold of MDA-MB-231
	**MDA-MB-231**	10520.995	44865.622	0.2345	1
	**RT-R-MDA-MB-231**	14580.288	35113.660	0.4152	1.7707
	**CD24-44+**	46347.522	52873.936	0.8766	3.7380

<Table S8: Summary of relative protein levels for Figure 2D>

LOX	MDA-MB-231	RT-R-MDA-MB-231	CD24-44+
n=1	1	3.6523	4.2001
n=2	1	1.6508	6.8779
n=3	1	3.1861	3.4179
n=4	1	1.4744	2.9702
n=5	1	1.7707	3.7380
Mean	1	2.3470	4.2410
**Std. Deviation**	**0**	**0.9983**	**1.5410**
**Std. Error of Mean**	**0**	**0.4464**	**0.6892**

<Table S9: Intensity ratios of Western blots for Figure 4B>

Fig 4B (MICA/B)	n=1	MICA/B	β-actin	MICA/B/β-actin	Fold of MDA-MB-231
	**MDA-MB-231**	38353.856	53827.936	0.7125	1
	**RT-R-MDA-MB-231**	18660.966	49602.652	0.3762	0.5280
	**CD24-44+**	28597.300	46618.894	0.6134	0.8609
	**n=2**	**MICA/B**	**β-actin**	**MICA/B/β-actin**	**Fold of MDA-MB-231**
	**MDA-MB-231**	48146.543	60901.744	0.7906	1
	**RT-R-MDA-MB-231**	44970.078	57929.539	0.7763	0.9819
	**CD24-44+**	36845.229	58088.530	0.6343	0.8023
	**n=3**	**MICA/B**	**β-actin**	**MICA/B/β-actin**	**Fold of MDA-MB-231**
	**MDA-MB-231**	41879.966	51066.057	0.8201	1
	**RT-R-MDA-MB-231**	14517.217	44000.673	0.3299	0.4023
	**CD24-44+**	22328.765	42318.167	0.5276	0.6434
	**n=4**	**MICA/B**	**β-actin**	**MICA/B/β-actin**	**Fold of MDA-MB-231**
	**MDA-MB-231**	43752.472	56742.530	0.7711	1
	**RT-R-MDA-MB-231**	20431.610	53170.380	0.3843	0.4984
	**CD24-44+**	24705.563	54667.853	0.4519	0.5861

	n=5	MICA/B	β-actin	MICA/B/β-actin	Fold of MDA-MB-231
	**MDA-MB-231**	38519.421	44997.794	0.8560	1
	**RT-R-MDA-MB-231**	29729.087	48119.087	0.6178	0.7217
	**CD24-44+**	33413.087	55532.652	0.6017	0.7029

**Fig 4B (HLA-E)**	**n=1**	**HLA-E**	**β-actin**	**HLA-E/β-actin**	**Fold of MDA-MB-231**
	**MDA-MB-231**	20532.066	66057.773	0.3108	1
	**RT-R-MDA-MB-231**	43185.915	56005.368	0.7711	2.4809
	**CD24-44+**	46312.501	52586.853	0.8807	2.8334
	**n=2**	**HLA-E**	**β-actin**	**HLA-E/β-actin**	**Fold of MDA-MB-231**
	**MDA-MB-231**	21484.501	54231.329	0.3962	1
	**RT-R-MDA-MB-231**	48170.451	53660.945	0.8977	2.2659
	**CD24-44+**	44237.986	47934.309	0.9229	2.3296
	**n=3**	**HLA-E**	**β-actin**	**HLA-E/β-actin**	**Fold of MDA-MB-231**
	**MDA-MB-231**	25823.108	50298.693	0.5134	1
	**RT-R-MDA-MB-231**	48141.158	46365.551	1.0383	2.0224
	**CD24-44+**	37334.602	35687.702	1.0461	2.0377
	**n=4**	**HLA-E**	**β-actin**	**HLA-E/β-actin**	**Fold of MDA-MB-231**

**MDA-MB-231**	5627.933	52122.167	0.1080	1
**RT-R-MDA-MB-231**	11154.560	50384.037	0.2214	2.0504
**CD24-44+**	11283.246	50160.045	0.2249	2.0833

n=5	HLA-E	β-actin	HLA-E/β-actin	Fold of MDA-MB-231
**MDA-MB-231**	28505.401	54339.673	0.5246	1
**RT-R-MDA-MB-231**	45096.300	50763.158	0.8884	1.6935
**CD24-44+**	35697.986	32983.652	1.0823	2.0632

<Table S10: Summary of relative protein levels for Figure 4B>

MICA/B	MDA-MB-231	RT-R-MDA-MB-231	CD24-44+
n=1	1	0.5280	0.8609
n=2	1	0.9819	0.8023
n=3	1	0.4023	0.6434
n=4	1	0.4984	0.5861
n=5	1	0.7217	0.7029
**Mean**	**1**	**0.6265**	**0.7191**
**Std. Deviation**	**0**	**0.2301**	**0.1126**
**Std. Error of Mean**	**0**	**0.1029**	**0.0504**

HLA-E	MDA-MB-231	RT-R-MDA-MB-231	CD24-44+

n=1	1	2.4809	2.8334
n=2	1	2.2659	2.3296
n=3	1	2.0224	2.0377
n=4	1	2.0504	2.0833
n=5	1	1.6935	2.0632
**Mean**	**1**	**2.1030**	**2.2690**
**Std. Deviation**	**0**	**0.2942**	**0.3364**
**Std. Error of Mean**	**0**	**0.1316**	**0.1504**

<Table S11: Intensity ratios of Western blots for Figure 4C>

**Fig 4C (pre-mature ADAM-10)**	**n=1**	**p-ADAM-10**	**β-actin**	**p-ADAM-10/β-actin**	**Fold of MDA-MB-231**
	**MDA-MB-231**	45249.472	56433.095	0.8018	1
	**RT-R-MDA-MB-231**	24984.007	61412.217	0.4068	0.5074
	**CD24-44+**	9900.681	53002.794	0.1868	0.2330
	**n=2**	**p-ADAM-10**	**β-actin**	**p-ADAM-10/β-actin**	**Fold of MDA-MB-231**
	**MDA-MB-231**	49301.572	56281.380	0.8760	1
	**RT-R-MDA-MB-231**	11563.924	46585.368	0.2482	0.2834
	**CD24-44+**	13601.388	57030.409	0.2385	0.2723
	**n=3**	**p-ADAM-10**	**β-actin**	**p-ADAM-10/β-actin**	**Fold of MDA-MB-231**
	**MDA-MB-231**	56018.907	60388.309	0.9276	1
	**RT-R-MDA-MB-231**	9534.137	48917.116	0.1949	0.2101
	**CD24-44+**	11368.338	51003.409	0.2229	0.2403
	**n=4**	**p-ADAM-10**	**β-actin**	**p-ADAM-10/β-actin**	**Fold of MDA-MB-231**
	**MDA-MB-231**	46340.765	57147.551	0.8109	1
	**RT-R-MDA-MB-231**	9247.459	52907.966	0.1748	0.2155
	**CD24-44+**	8979.388	40626.459	0.2210	0.2726

	n=5	p-ADAM-10	β-actin	p-ADAM-10/β-actin	Fold of MDA-MB-231
	**MDA-MB-231**	21175.794	56740.915	0.3732	1
	**RT-R-MDA-MB-231**	2999.335	50194.915	0.0598	0.1601
	**CD24-44+**	3579.891	53260.480	0.0672	0.1801

**Fig 4C (mature ADAM-10)**	**n=1**	**m-ADAM-10**	**β-actin**	**m-ADAM-10/β-actin**	**Fold of MDA-MB-231**
	**MDA-MB-231**	12552.631	56433.095	0.2224	1
	**RT-R-MDA-MB-231**	37451.472	61412.217	0.6098	2.7417
	**CD24-44+**	30347.823	53002.794	0.5726	2.5741
	**n=2**	**m-ADAM-10**	**β-actin**	**m-ADAM-10/β-actin**	**Fold of MDA-MB-231**
	**MDA-MB-231**	22682.108	56281.380	0.4030	1
	**RT-R-MDA-MB-231**	49141.936	46585.368	1.0549	2.6175
	**CD24-44+**	51244.794	57030.409	0.8986	2.2296
	**n=3**	**m-ADAM-10**	**β-actin**	**m-ADAM-10/β-actin**	**Fold of MDA-MB-231**
	**MDA-MB-231**	28637.279	60388.309	0.4742	1
	**RT-R-MDA-MB-231**	48441.179	48917.116	0.9903	2.0882
	**CD24-44+**	47892.451	51003.409	0.9390	1.9801
	**n=4**	**m-ADAM-10**	**β-actin**	**m-ADAM-10/β-actin**	**Fold of MDA-MB-231**

	**MDA-MB-231**	29686.279	57147.551	0.5195	1
	**RT-R-MDA-MB-231**	48283.936	52907.966	0.9126	1.7568
	**CD24-44+**	52207.622	40626.459	1.2851	2.4738
	**n=5**	**m-ADAM-10**	**β-actin**	**m-ADAM-10/β-actin**	**Fold of MDA-MB-231**
	**MDA-MB-231**	23805.794	56740.915	0.4196	1
	**RT-R-MDA-MB-231**	28205.128	50194.915	0.5619	1.3393
	**CD24-44+**	48041.279	53260.480	0.9020	2.1499

<Table S12: Summary of relative protein levels for Figure 4C>

p-ADAM-10	MDA-MB-231	RT-R-MDA-MB-231	CD24-44+
n=1	1	0.5074	0.2330
n=2	1	0.2834	0.2723
n=3	1	0.2101	0.2403
n=4	1	0.2155	0.2726
n=5	1	0.1601	0.1801
**Mean**	**1**	**0.2753**	**0.2396**
**Std. Deviation**	**0**	**0.1369**	**0.0379**
**Std. Error of Mean**	**0**	**0.0612**	**0.0169**

m-ADAM-10	MDA-MB-231	RT-R-MDA-MB-231	CD24-44+

n=1	1	2.7417	2.5741
n=2	1	2.6175	2.2296
n=3	1	2.0882	1.9801
n=4	1	1.7568	2.4738
n=5	1	1.3393	2.1499
**Mean**	**1**	**2.1090**	**2.2820**
**Std. Deviation**	**0**	**0.5865**	**0.2416**
**Std. Error of Mean**	**0**	**0.2623**	**0.1080**
